# Assessing cultivar-specific susceptibility and morphological adaptations of *Bactrocera oleae* (Diptera: Tephritidae) in olive orchards

**DOI:** 10.1093/jisesa/ieaf005

**Published:** 2025-02-12

**Authors:** Ivana Pajač Živković, Dana Čirjak, Luka Hojsak, Barbara Vrček, Manuel J Suazo, Hugo A Benitez, Darija Lemic

**Affiliations:** Department of Agricultural Zoology, University of Zagreb Faculty of Agriculture, Zagreb, Croatia; Department of Agricultural Zoology, University of Zagreb Faculty of Agriculture, Zagreb, Croatia; Department of Agricultural Zoology, University of Zagreb Faculty of Agriculture, Zagreb, Croatia; Department of Agricultural Zoology, University of Zagreb Faculty of Agriculture, Zagreb, Croatia; Instituto de Alta Investigación, Universidad de Tarapacá, Arica, Chile; Laboratorio de Ecología y Morfometría Evolutiva, Centro de Investigación de Estudios Avanzados del Maule, Universidad Católica del Maule, Talca, Chile; Centro de Investigación en Recursos Naturales y Sustentabilidad (CIRENYS), Universidad Bernardo O’Higgins, Santiago, Chile; Cape Horn International Center (CHIC), Centro Universitario Cabo de Hornos, Universidad de Magallanes, Puerto Williams, Chile; Department of Agricultural Zoology, University of Zagreb Faculty of Agriculture, Zagreb, Croatia

**Keywords:** Tephritidae, cultivar susceptibility, geometric morphometrics, wing shape adaptation

## Abstract

The olive fruit fly, *Bactrocera oleae* (Rossi), is the most important widespread pest in olive-growing areas worldwide, causing significant yield losses and deterioration of olive oil quality. This study hypothesized that olive cultivars differ in their susceptibility to *B.oleae* and that the cultivar in which the pest develops may affect population variability. The primary goal was to assess susceptibility across 6 olive cultivars, while the secondary goal was to analyze population variability using geometric morphometrics to identify cultivar-specific phenotypic differences. Conducted at 2 sites, Banjevci (Dalmatia) and Vodnjan (Istria), the study revealed significant differences in infestation levels, emergence, and wing morphology. Higher humidity in Vodnjan favored increased fly populations. Larger-fruited cultivars such as Oblica, Istarska bjelica, and Ascolana tenera experienced higher infestation rates, whereas smaller-fruited cultivars like Frantoio and Leccino exhibited lower infestation levels. Wing shape analysis highlighted sexual dimorphism, with female flies exhibiting broader wings that may enhance dispersal and oviposition efficiency, particularly in larger fruits. The observed phenotypic plasticity of *B. oleae* across cultivars and locations indicates an ability to adapt to varied host and cultivation conditions, enhancing pest resilience. The findings underscore the role of cultivar selection as a passive pest management strategy and demonstrate the utility of geometric morphometrics in capturing cultivar-specific morphological adaptations.

## Introduction

The cultivation of the olive (*Olea europaea* L.) has been present in the Mediterranean region for more than 2,500 years. It is a cornerstone of the agricultural heritage and of great historical and economic importance (Žužić 2002). This economically important crop is constantly threatened by various pests, of which the olive fruit fly, *Bactrocera oleae* (Rossi) (Diptera: Tephritidae), is the main pest responsible for considerable yield losses and a deterioration in olive oil quality (Maceljski 2002). *Bactrocera oleae* is widespread in olive-growing areas worldwide, from the Mediterranean to southern Africa, California, Hawaii, and Mexico (Brnetić 1979, EPPO 2024).

*Bactocera oleae* causes damage by laying eggs in the olive fruit. After hatching, the larvae feed on the mesocarp of the fruit and cause mechanical destruction of the plant tissue (Podgornik et al. 2013, Dminić et al. 2015). By feeding on olive fruit, *B. oleae* causes premature fruit drop (20% to 80% loss), reduced fruit weight (50 to 270 mg/olive), reduced oil content (10% to 15%), and oxidative degradation of phenolic compounds, which impairs oil quality (Šindrak et al. 2007, Dminić et al. 2015). The data collected by Malheiro et al. (2015) showed that *B. oleae* infestation impairs the chemical, sensory, and nutritional properties of olive oil. The development of integrated pest management (IPM) approaches offers sustainable alternatives that integrate biological control, cultural practices, and many other non-chemical measures (Daane and Johnson 2010). Understanding the complex interactions between *B. oleae* and olive cultivars is necessary to develop effective IPM strategies that are adapted to local conditions and cultivar characteristics.

Olive cultivars differ in susceptibility to *B. oleae* (Garantonakis et al. 2016). Previous studies have shown that factors such as fruit shape, size and weight, color, flesh firmness, surface coverage, phenological stage, and chemical composition influence the degree of susceptibility to infestation by *B. oleae* (Daane and Johnson 2010, Karakoyun and Uçkun 2023). Cultivars with spherical and larger fruits, low epicarp hardness, thinner wax layers, and late ripening (green color until the end of the season) are more susceptible to infestation and more attractive to female *B. oleae* for oviposition (Daane and Johnson 2010, Karakoyun and Uçkun 2023).

The survival and development of *B. oleae* in the Mediterranean regions depend on local weather conditions, with temperature and relative humidity being the most important (Preu et al. 2020). Low mortality rates are observed in mild winters, while high mortality occurs at extreme temperatures, with significant losses at high summer temperatures and low winter temperatures. Therefore, the temperature conditions of previous seasons are necessary for determining the infestation rate (Marchi et al. 2016, Preu et al. 2020). In addition, survival, longevity, and maturation are strongly correlated with relative humidity (Broufas et al. 2009, Preu et al. 2020), with high humidity increasing the risk of *B. oleae* infestation (Gratsea et al. 2022).

In addition to examining weather conditions and organoleptic characteristics of olive cultivars, it is important to investigate the potential variability of *B. oleae* populations based on its preferred olive cultivars. Wing shape and size, analyzed through geometric morphometrics, are among the first morphological traits to exhibit changes, as they reflect both environmental and genetic influences (Bouyer et al. 2007). This makes them useful for detecting and monitoring population variations, providing a tool for assessing adaptive responses in different populations (Mikac et al. 2013, Benitez et al. 2014a). This tool provides data by measuring the distance between certain points (markers) placed at the crossing points of the wing veins (Klingenberg 2010). Due to its low cost and rapid application, it is a valuable tool for understanding phenotypic variability and population structure (Klingenberg 2013). Studies on geometric morphometrics in different insect species have shown that morphological variation is related to behavior, dispersal, biological adaptation, and responses to environmental stress (Mikac et al. 2019, Pajač Živković et al. 2019, Lemic et al. 2020). Geometric morphometrics has been successfully used to study various body parts of different insect species, in particular the wings (Breuker et al. 2010, Benítez et al. 2014b, Mikac et al. 2016, Lemic et al. 2021a) and of pests from the order Diptera (de Souza et al. 2015, Pieterse et al. 2017, Pajač Živković et al. 2018, Lemic et al. 2020, 2021b). Wing shape has proven to be an effective trait for measurement in various agronomic and ecology studies, including analyses of life-history traits such as sexual dimorphism and both interspecific and intraspecific shape variation (Brisson 2010, Camargo and de Oliveira 2012, Benitez et al. 2014a, Lemic et al. 2014, Reis et al. 2021).

In this study, we hypothesized that olive cultivars exhibit differential susceptibility to *B. oleae* infestation and that the specific cultivar in which *B. oleae* develops may drive variability within the pest population. The primary objective of this study was to assess the susceptibility of 6 distinct olive cultivars to *B. oleae* infestation. The secondary objective was to evaluate population variability by analyzing *B. oleae* wing shape across cultivars, using geometric morphometric techniques to quantify phenotypic differences linked to cultivar-specific development conditions.

## Material and Methods

### Study Sites and Olive Cultivars

**Fig. 1.**
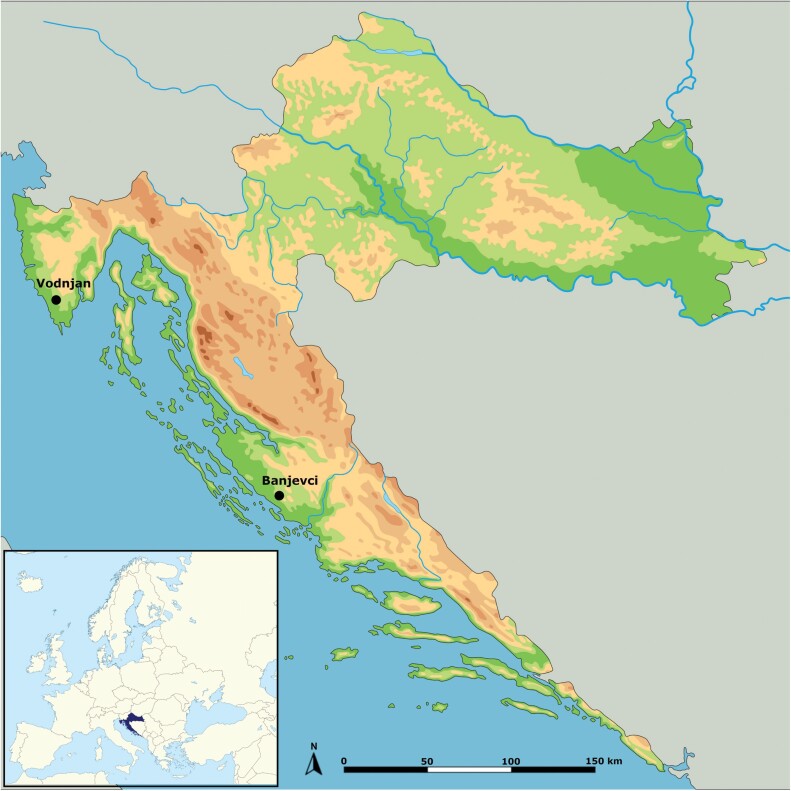
Coastal locations in Croatia where *Bactrocera oleae* was collected on fruits of indigenous and domesticated olive cultivars (under the terms of the Shutterstock license and created with GIMP 2.10.2 software).

The study on olive susceptibility and morphological variability of *B. oleae* on native and domesticated olive cultivars was conducted in Croatian coastal olive groves in central Dalmatia and Istria. The Central Dalmatian growing area was represented by the olive grove of a family farm in Banjevci (43°54ʹ59.9 ″N 15°36ʹ50.2 ″E) and the Istrian growing area was represented by the Croatian National Collection of Domesticated Olive Varieties in Vodnjan (44°58ʹ24.2 ″N 13°51ʹ02.5 ″E) (Fig. 1.). In Banjevci, 7 olive cultivars were cultivated on a 7.5-hectare area following organic farming principles, using biological insecticides based on *Bacillus thuringiensis* subsp. *kurstaki* and foliar applications of kaolin for pest control. In Vodnjan, 58 olive cultivars were grown on a 2.5-hectare area under integrated farming principles, currently transitioning from integrated to organic farming. To enhance plant defenses, biostimulants (organic nitrogen fertilizers) were applied, along with permitted insecticides for IPM and kaolin for pest control. Organic farming in Banjevci relies entirely on natural inputs for pest and nutrient management, while integrated farming in Vodnjan combines organic practices with regulated, limited use of synthetic inputs.

In the 2020 harvest season (from 25 October to 7 November), olive fruits of the same 6 cultivars (2 indigenous—Oblica and Istarska bjelica and 4 domesticated—Ascolana tenera, Frantoio, Leccino, and Pendolino) were harvested in Vodnjan and Banjevci. The ripening times of the olive cultivars studied vary slightly, reflecting differences in adaptation to local climatic conditions and micro-locations. Istarska bjelica is an early-ripening cultivar, typically ready for harvest in early October; Ascolana tenera is an early to mid-early variety that ripens later in Croatia than in its native Italy, reaching maturity in October or early November; Leccino, also classified as early to mid-early, usually ripens mid-to-late October, while Frantoio ripens mid-to-late season, generally from October through early November, slightly earlier than some native cultivars; Pendolino cultivar ripens between mid-October and early November while Oblica, a mid-late variety, extends its ripening from late October through early December (Bulimbašić 2011).

Three olive trees of each listed cultivar were sampled. One hundred olive fruits were collected per tree, i.e., 25 fruits each from different canopy heights, from the northern, southern, eastern, and western parts of the tree, using the method of Burrack and Zalom (2008).

The sampled fruits were taken to the entomological laboratory of the Faculty of Agriculture Zagreb, where they were weighed and visually examined to separate the uninfested fruits from the infested ones based on the scars caused by the oviposition of *B. oleae* females and to estimate the percentage of infested fruits per cultivar. The olive fruits (uninfested separated from infested) were then sorted by cultivar into 1 litre plastic buckets containing a 5-cm layer of coarse Vermiculite to absorb moisture during fruit decay. Each bucket of olive samples was then sealed in a clear plastic bag with a capacity of 6 liters, perforated with about 100 pinholes to reduce condensation. The containers were set up on 9 November and monitored while all flies were developing (until 11 January). The developing flies were counted and preserved in 70% EtOH for identification. Identification of the collected species and sex was based on standard morphological characteristics (Byron and Gillett-Kaufman 1999, Barić and Pajač 2012).

### Weather Conditions

The data used in this study (i.e., mean air temperature, total monthly precipitation, and mean daily relative humidity for 2020) were obtained from the Croatian Meteorological and Hydrological Service for the year of sampling and analyzed for each field site. The maximum distance between the meteorological stations and the olive orchards was 20 km. Data on weather conditions were subjected to analysis of variance (ANOVA). We performed ANOVA on the monthly weather condition data (mean air temperature, total monthly precipitation, and mean daily relative humidity) to compare differences across locations.

### Cultivar Susceptibility Analysis

The number of flies in each replicate of each cultivar was recorded throughout the study period and calculated based on the average number of flies per cultivar. ANOVA was performed on the mean number of flies to determine their cultivar susceptibility according to cultivar and location. ANOVA was also conducted on the weight of 100 fruits and the ratio of healthy to infested fruits. If significant differences were found, a post-hoc test of means was performed (Tukey’s HSD).

Statistical data processing (ANOVA, Tukey’s HSD test) was performed using ARM 2019 GDM software.

### Multivariate Analysis of Shape

The left and right wings of each fly were removed and fixed laterally with Euparal fixative (Carl Roth GmbH + Co. KG, Karlsruhe, Germany) according to standard methods (Upton and Mantel 2010) for subsequent morphometric analysis. A total of 453 (217 males, 236 females) *B. oleae* from Vodnjan and 211 (109 males, 102 females) *B. oleae* from Banjevci were included in the study (Table 1).

**Table 1.** Representation of *Bactrocera oleae* populations used for morphometric analyses sorted by location and olive cultivar

Location	Olive variety	Males *Bactrocera oleae*	Females *Bactrocera oleae*	Population (*n*)
Banjevci	Oblica	14	8	22
	Istarska bjelica	19	18	37
	Ascolana tenera	14	25	39
	Frantoio	6	8	14
	Leccino	20	27	47
	Pendolino	21	11	33
Vodnjan	Oblica	24	42	66
	Istarska bjelica	44	43	87
	Ascolana tenera	27	41	68
	Frantoio	43	46	89
	Leccino	33	29	62
	Pendolino	40	31	71

**Fig. 2.**
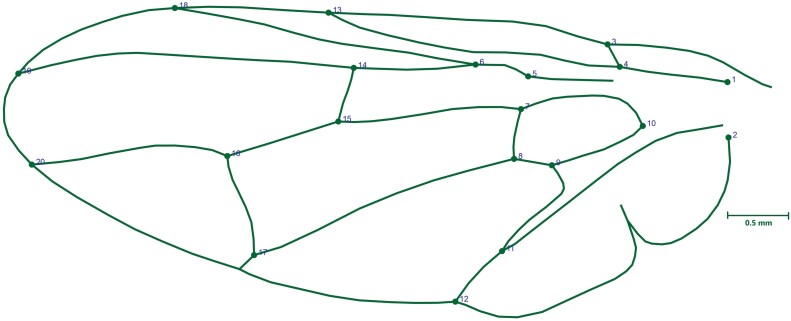
Graphical representation of the Wing shape of *Bactrocera oleae* with 20 landmarks.

A total of 664 *B. oleae* (326 males + 338 females) had their wings photographed with a Nikon D780 camera. Each wing was mounted and photographed individually. The images were saved in jpg format and organized according to their respective positions. The images were converted to tps format using TPSUtil v1.81 software. Subsequently, 20 landmarks (see Fig. 2) were digitized at the intersections and edges of the tree longitudinal veins (from anterior to posterior): Radius—landmarks 3, 13, 18, and 19; Media—landmarks 1, 4, 5, 6, 7, 8, 9, 10, 14, 15, 16, 17, and 20 and Anal—landmarks 2, 11, and 12 using the software tpsDig2 v2.31 (Rohlf 2008). Wings with damaged areas preventing proper landmark placement were excluded from the analysis.

Cartesian coordinates were determined for all landmarks and the shape information was extracted using a Generalized Procrustes Analysis (Rohlf and Slice 1990). This Procrustes superimposition procedure standardizes each specimen by removing size, position, and orientation information and adjusting it according to the size of the centroid. The shape space of wing shape variation was projected by ordinal principal component analysis (PCA) using the covariance matrix of individuals. The mean shape was projected using a covariance matrix of averaged locations and extracted and superimposed to identify landmark variation between groups.

A canonical analysis of variance between the sexes and the sites was performed to graphically represent the differences in shape, and a permutation test (1,000 rounds) was performed to determine statistical differences between the sites. Finally, a multivariate regression was performed to determine differences between sexes using centroid size as the independent variable and shape as the dependent variable.

## Results

### Weather Conditions

There were no statistically significant differences in the mean air temperatures (*F*_1,22_ = 0.06; *P* = 0.79) and the total amount of precipitation (*F*_1,22_ = 0.02; *P* = 0.88) between the analyzed sites. However, significant differences were found in mean daily relative humidity (*F*_1,22_ = 5.15; *P* = 0.03). The ANOVA analysis of the monthly weather data showed that the humidity at the Vodnjan site was significantly higher than at the Banjevci site. The average daily relative humidity (%) was between 6 and 12 % (depending on the month) higher in Vodnjan than in Banjevci each month. These variations in humidity could affect the local microclimate and potentially influence site-specific environmental conditions and agricultural outcomes. Details of all observed weather parameters during the study period, including temperature, precipitation, and humidity, can be found in Table 2.

**Table 2.** Weather conditions on two study locations during 2020

Month	Mean monthly temperature (^◦^C)		Monthly amount of precipitation (mm)		Average daily relative humidity (%)	
	Vodnjan	Banjevci	Vodnjan	Banjevci	Vodnjan	Banjevci
January	7.4	5.3	13.4	21.6	71	81
February	9.5	8.4	8.1	22.5	70	77
March	9.9	9.4	47.5	40.0	60	68
April	14.3	13.4	3.6	14.8	54	58
May	18.8	18.3	14.3	21.4	53	65
June	22.2	21.4	69.7	160.7	63	72
July	24.7	24.8	41.7	5.2	52	64
August	25.5	25.6	44.7	46.3	59	71
September	21.0	20.7	201.8	133.4	66	73
October	14.5	13.7	180.5	208.3	79	87
November	11.0	9.4	38.9	45.6	74	80
December	8.9	8.7	200.5	201.3	78	87

The monthly precipitation data has shown that the 2 locations have very similar thermal conditions. Both Vodnjan and Banjevci showed an increase in precipitation in June and then again in September, October, and December. This indicates a pattern that is probably related to the regional climatic conditions that affect both areas in summer, autumn, and early winter. The lowest rainfall in both locations occurred at the beginning of the year, which is typical of Mediterranean climates with dry summers and wet winters. The temperature for Vodnjan and Banjevci followed similar trajectories, with the highest temperatures occurring in the middle of the year, around July, and the lowest in the winter months of January and December. We found that Vodnjan has a higher relative humidity than Banjevci throughout the year. The higher humidity in Vodnjan could be influenced by local geographical conditions such as proximity to the sea, higher local vegetation density or prevailing winds that transport moisture. The data provided can be considered largely representative of the region’s general climatic trends (DHMZ 2024).

### Host Susceptibility

The emergence of *B. oleae* in Vodnjan began on 9 November and lasted until 11 January. In Banjevci, the emergence of flies began on 16 November and lasted until 21 December. During the emergence period, a total of 687 specimens of *B. oleae* developed on olive fruit in Vodnjan, 215 of them on visually uninfested fruit and 472 on visually infested fruit. In Banjevci, 88 specimens developed from visually uninfested fruits, while 124 specimens emerged from visually infested fruits, totaling 212 *B. oleae* specimens from this area. In Vodnjan, the Indigenous cultivar Istarska bjelica had the highest number of developed flies (145), followed by Frantoio (118) and Ascolana tenera (79), while the lowest development was observed in Leccino (70). In Banjevci, the highest number of *B. oleae* flies developed in the Ascolana tenera cultivar (55), followed by Leccino (47) and Istarska bjelica (39), while the lowest development was recorded in Frantoio (12) (details in Table 3). For most varieties, a higher percentage of flies developed from visually infested fruits. For instance, in Vodnjan, Oblica and Istarska bjelica had 78% and 69% developed flies from infested fruits, respectively. In Banjevci, this pattern is held with high percentages from infested fruits in varieties like Ascolana tenera (82%) and Oblica (65%). Exceptions include Leccino in Vodnjan and Banjevci, where 53% and 72% of flies developed from visually uninfested fruits, indicating possible differences in how infestation is visually assessed or variations in pest development in uninfested fruits.

**Table 3.** Period of emergence of *Bactrocera oleae* flies on the collected fruits, ratio of visually uninfested and infested fruits, with the total number (and ratio) of developed flies on visually uninfested and infested fruits, sorted by location and olive cultivar

Location	Variety	Period of occurrence of the flies from fruits	Ratio of fruit per sample (%)		Total number of developed flies		Ratio of developed flies (%)	
			Visually uninfested	Visually infested	Visually uninfested fruits	Visually infested fruits	Visually uninfested fruits	Visually infested fruits
Vodnjan	Istarska bjelica	November 30 to January11	19	91	45	100	31	69
	Oblica	November 9 to January 11	12	88	17	59	22	78
	Ascolana tenera	December 4 to January 11	27	73	37	42	47	53
	Frantoio	November 9 to January 8	23	77	44	74	37	63
	Leccino	November 13 to January 8	30	70	37	33	53	47
	Pendolino	December 4 to January 8	44	56	35	40	47	53
Banjevci	Istarska bjelica	November 16 to December 7	80	20	14	25	36	64
	Oblica	November 16 to December 21	54	46	9	17	35	65
	Ascolana tenera	November 16 to December 21	48	52	10	45	18	82
	Frantoio	December 4 to December 21	87	13	5	7	42	58
	Leccino	November 20 to December 21	85	15	34	13	72	28
	Pendolino	December 11 to December 21	75	25	16	17	48	52

In Table 4, the data from the Vodnjan site show that the Oblica and Ascolana tenera cultivars produced the higher fruit weight, while the Pendolino and Leccino cultivars yielded the lower fruit weight. Visual inspections show that the Oblica cultivar suffered the most damage from oviposition, while the Pendolino cultivar experienced the least damage to the fruit.

**Table 4.** ANOVA of average weight of 100 fruits (± SE) and the average number (± SE) of infested and uninfested fruits for olive cultivars in the Vodnjan determined, by visual inspection

Cultivar	Weight of 100 fruits [g]	Average No. infested fruits	Average No. uninfested fruits
Ascolana tenera	479.8 ± 8.0 a^*^	73.0 ± 1.5 b	27.0 ± 1.5 b
Oblica	489.3 ± 9.8 a	85.5 ± 3.5 a	14.5 ± 3.5 c
Pendolino	175 ± 0.0 c	55.0 ± 2.5 c	45.0 ± 2.5 a
Frantoio	262.8 ± 4.6 b	76.5 ± 2.3 ab	23.5 ± 2.3 bc
Leccino	192.3 ± 2.7 c	70.3 ± 2.1 b	29.8 ± 2.1 b
Istarska bjelica	260.5 ± 3.7 b	81.0 ± 2.7 ab	19.0 ± 2.7 bc
Tukey’s HSD ** *P* = 0.05	23.42	12.23	12.23

^*^values within a column marked with the same letter do not differ significantly (p > 0.05; HSD test); ** HSD was determined by comparing the weight of 100 fruits, average number of infested and uninfested fruits.

In Table 5, the data from the Banjevci site show that the Oblica cultivar produced the higher fruit weight, closely followed by Ascolana tenera, while the lower fruit weight was developed by the Frantoio and Istarska bjelica cultivars. Visual inspections showed that the Oblica and Ascolana tenera cultivars were the most severely damaged by oviposition, while the Frantoio cultivar had the least fruit damage.

**Table 5.** ANOVA of average weight of 100 fruits (± SE) and the average number (± SE) of infested and uninfested fruits for olive cultivars in the Banjevci determined, by visual inspection

Cultivar	Weight of 100 fruits [g]	Average No. infested fruits	Average No. uninfested fruits
Ascolana tenera	458.3 ± 4.4 a^*^	53.0 ± 2.7 a	47.0 ± 2.7 c
Oblica	479.5 ± 7.5 a	45.8 ± 2.3 a	54.3 ± 2.3 c
Pendolino	165.0 ± 0.0 b	24.0 ± 2.3 b	76.0 ± 2.3 b
Frantoio	142.3 ± 3.2 c	13.3 ± 1.0 c	86.8 ± 1.0 a
Leccino	173.0 ± 8.0 b	15.5 ± 1.4 bc	84.5 ± 1.4 ab
Istarska bjelica	132.5 ± 3.4 c	20.8 ± 1.7 bc	79.3 ± 1.7 ab
Tukey’s HSD ** *P* = 0.05	21.28	8.74	8.74

^*^Values within a column marked with the same letter do not differ significantly (*P* > 0.05; HSD test); ** HSD was determined by comparing the weight of 100 fruits, the average number of infested and uninfested fruits.

Table 6 shows the results of the ANOVA between the average number of developed *B. oleae* on the tested cultivars depending on the tested location during the entire harvest period. In Vodnjan, Istarska bjelica had the highest mean number of developed flies (48.0), significantly different from other cultivars, suggesting higher susceptibility in this location. In contrast, Frantoio showed fewer developed flies (36.8), and other cultivars like Pendolino, Ascolana tenera, and Oblica had lower but similar numbers, with no significant differences among them. In Banjevci, the number of developed flies was generally lower for all cultivars, with Ascolana tenera and Leccino having higher values but still significantly lower than their counterparts in Vodnjan. This trend shows that Banjevci generally had lower fly development across all cultivars, which may suggest environmental factors or location-specific characteristics affecting fly development.

**Table 6.** ANOVA of Mean Number (± SE) of total emerged olive flies (per 100 fruits) across different cultivars and locations

Cultivar	Vodnjan	Banjevci	HSD P = 0.05^+^
Ascolana tenera	24.8 ± 1.7 c A	19.3 ± 1.3 a* B^*^	4.2
Oblica	25.8 ± 2.3 c A	7.8 ± 1.7 cd B	9.8
Pendolino	22.8 ± 1.3 c A	11.0 ± 1.5 bc B	3.5
Frantoio	36.8 ± 2.7 b A	3.5 ± 1.0 d B	5.5
Leccino	22.0 ± 2.7 c A	14.5 ± 1.7 ab A	13.0
Istarska bjelica	48.0 ± 2.2 a A	12.0 ± 1.6 bc B	4.6
HSD *P* = 0.05**	10.4	6.6	

^*^Values marked with the same letter do not differ significantly (*P* > 0.05; HSD test); ** HSD was determined by comparing the average numbers of flies per cultivar; ^+^ HSD was determined by comparing the average numbers of flies per location.

### Wing Shape Variability

**Fig. 3.**
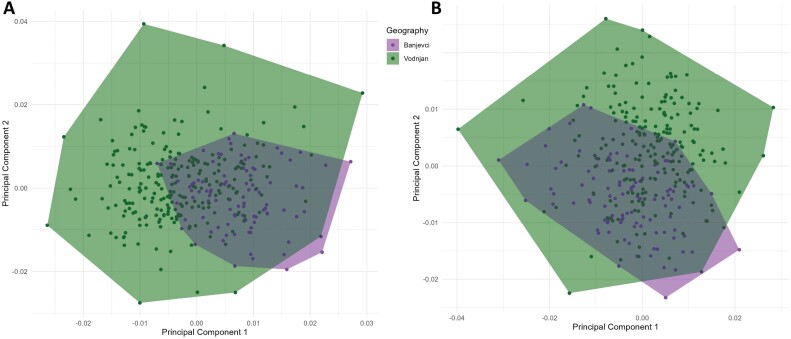
Principal component analysis of wing shape of *Bactrocera oleae* for both A: Females and B: Males between geographical location represented by purple: Banjevci and green: Vodnjan.

Our result revealed a strong component of sexual shape dimorphism on the fly wings, and we decided to analyze the wings separately by sex in order not to bias the results. PCA revealed separate differences in wing shape for female and male individuals between the localities Banjevci and Vodnjan. The PCA of females accumulated 37.5% of the variance in the first 3 components (PC1: 14.8%, PC2: 12.6%, PC3: 10.05%), and for males a similar percentage of 37.1% of the first 3 components (PC1:14.6%, PC2: 13.1%, PC3: 9.2%). For both plots, the wider variation in Vodnjan is noticeable, where individuals mostly utilize the entire morphospace of possible wing shape morphologies, in contrast to the more conserved morphology observed in Banjevci individuals, see Fig. 3 for both plots.

**Fig. 4.**
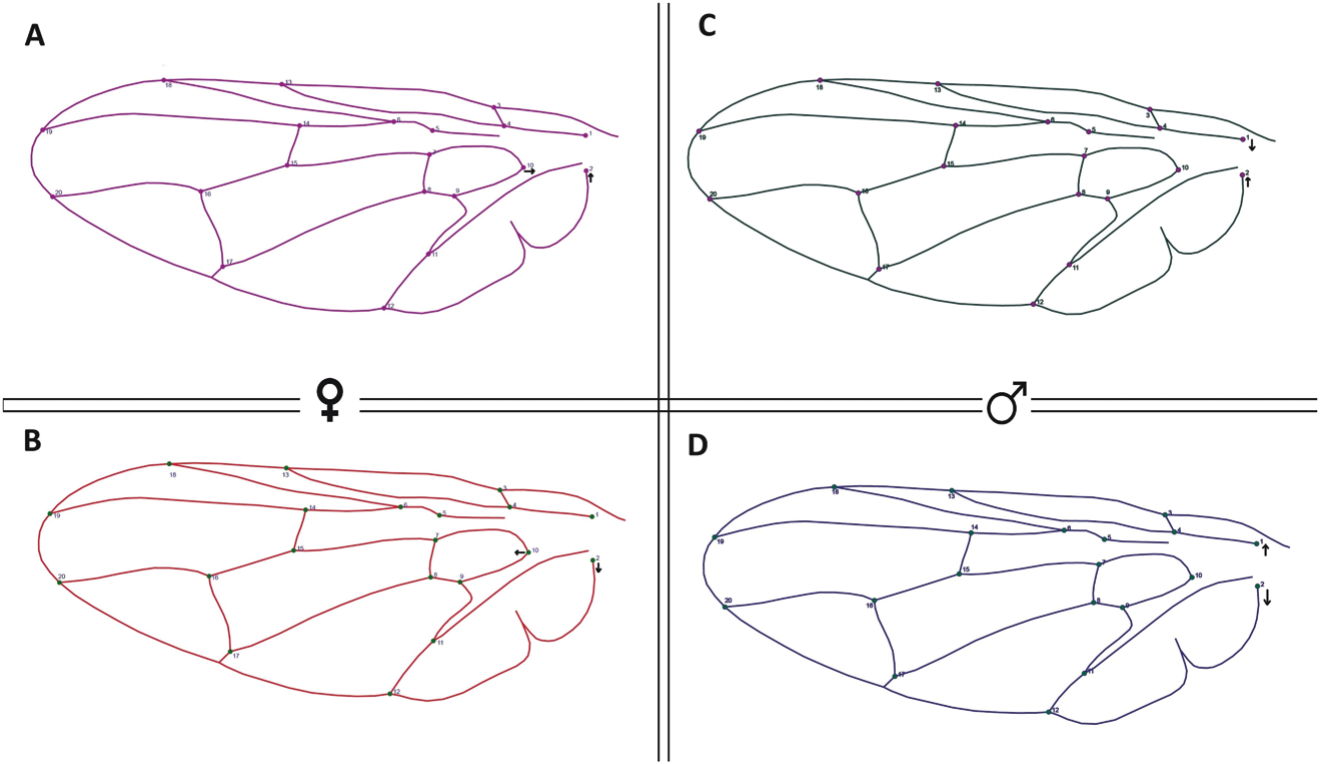
Average shape between males and females differenced by geographical location. Colors represent sex and cultivars: A: Purple females from Banjevci, B: Red females from Vodnjan, C: Green males from Banjevci, D: Blue males from Vodnjan.

The average shape between geographic locations shows an initial difference between males and females (Fig. 4), with landmark 12 contracting the wings of males and females, suggesting sexual shape dimorphism, as the female wings are wider than those of males. The variability of wings was found in both sexes in the Vodnjan population.

A canonical variate analysis (CVA) was performed to distinguish between the populations and to take a closer look at 6 cultivars from Vodnjan and the same 6 cultivars from Banjevci.

**Fig. 5.**
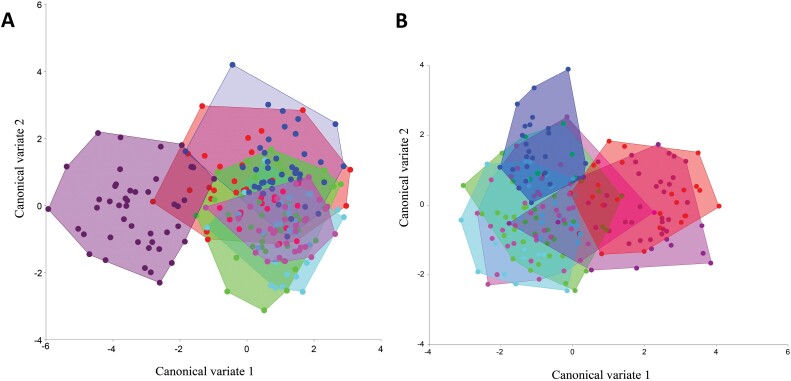
Canonical Variate analysis of the wing shape of *Bactrocera oleae* (for both A: Females and B: Males) from the Vodnjan site and their 6 cultivars represented by different colors: blue: Oblica, red: Ascolana tenera, dark red: Frantoio, green: Istarska bjelica, lightblue: Leccino, pink: Pendolino

The CVA of the females from the Vodnjan site showed a clear differentiation for the Frantoio cultivar, while the other cultivars seem to overlap, with some differences in Oblica and Istarska bjelica, which show a greater variance between cultivars. Among males, individuals of Frantoio did not vary as much compared to females, but they shared variation with more individuals of Ascolana tenera. Oblica appeared to differ from the other groups with less variation (Fig. 5). Table 7 shows Mahalanobis distances in wing shape among olive cultivars in Vodnjan, with variations observed between males and females. For females, Ascolana tenera and Frantoio stand out with significant differences, especially compared to cultivars like Leccino and Istarska bjelica. Frantoio has the most distinct wing shape for females, differing significantly from other cultivars. Pendolino also shows strong differences, particularly when compared with Frantoio and Oblica. Among males, differences in wing shape are less pronounced, with only a few cultivars, like Istarska bjelica and Leccino, showing significant distinctions. Overall, females show greater variation in wing shape across cultivars than males, indicating that wing shape differences may be more consistent among female flies (Table 7).

**Table 7.** Morphometric Mahalanobis Distances Between Multiple Locations in Vodnjan, Differentiated by Sex, with Respective Permutation Comparison p-values.

	CVA Females Vodnjan					CVA Males Vodnjan				
Mahalanobis Distances p-value	Ascolana tenera	Frantoio	Istarska bjelica	Leccino	Oblica	Ascolana tenera	Frantoio	Istarska bjelica	Leccino	Oblica
Frantoio	3.57 <0.0001					1.71 0.12				
Istarska bjelica	1.65 0.0076	4.19 <0.0001				2.92 <0.0001	3.02 < 0.0001			
Leccino	2.10 <0.0001	4.48 <0.0001	1.48 0.27			3.2 <0.0001	3.13 <0.0001	1.73 0.004		
Oblica	1.90 0.0001	4.43 <0.0001	2.01 <0.0001	2.09 <0.0001		3.36 <0.0001	3.45 <0.0001	2.22 <0.0001	2.23 <0.0001	
Pendolino	2.29 <0.0001	4.59 <0.0001	2.06 <0.0001	1.55 0.30	2.10 < 0.0001	3.05 <0.0001	3.03 <0.0001	1.53 0.056	1.29 0.65	2.10 0.0003

**Fig. 6.**
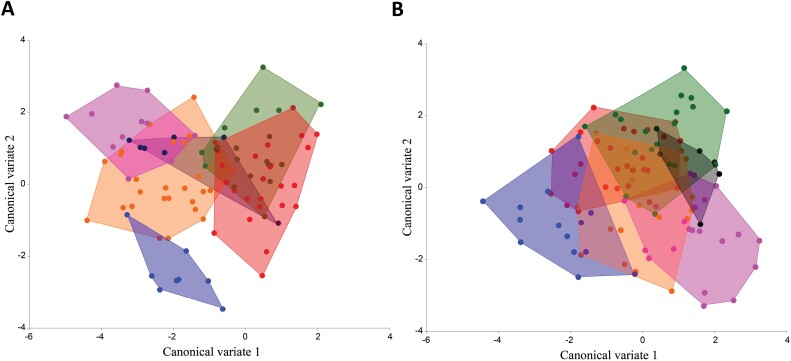
Canonical Variate analysis of the wing shape of *Bactrocera oleae* (for both A: Females and B: Males) from the Banjevci site and their 6 cultivars represented by different colors: blue: Oblica, red: Ascolana tenera, dark blue: Frantoio, green: Istarska bjelica, orange: Leccino, purple: Pendolino

Figure 6 shows the CVA for the cultivars of the Banjevci site, with the individuals from Oblica showing a greater disparity in females than the other cultivars. The individuals of Ascolana tenera differed from all other cultivars but had a certain overlap with Istarska bjelica. The situation was similar with the Leccino and Pendolino cultivars. On the other hand, the wing shape of Frantoio varied between the different groups. In the males, the variation in wing shape was less pronounced, with clear overlap among cultivars. The Mahalanobis distances showed clear differences when the cultivars were compared to each other. Only Istarska bjelica and Ascolana tenera, for females, were found to be non-significant in their wing shape (Table 8).

**Table 8.** Morphometric Mahalanobis Distances Between Multiple Locations in Banjevci, Differentiated by Sex, with Respective Permutation Comparison p-values.

	CVA Females Banjevci					CVA Males Banjevci				
Mahalanobis Distances P-value	Ascolana tenera	Frantoio	Istarska bjelica	Leccino	Oblica	Ascolana tenera	Frantoio	Istarska bjelica	Leccino	Oblica
Frantoio	3.58 <0.0001					3.05 0.008				
Istarska bjelica	1.76 0.68	3.25 0.004				2.64 0.007	3.28 0.01			
Leccino	3.15 <0.0001	2.99 0.003	2.97 <0.0001			2.22 0.007	2.97 0.007	2.85 0.01		
Oblica	3.90 <0.0001	4.28 0.0004	4.41 <0.0001	3.56 <0.0001		3.04 0.007	4.69 0.01	3.85 0.01	3.08 0.009	
Pendolino	4.34 <0.0001	3.77 0.0027	4.08 <0.0001	3.26 <0.0001	4.54 0.0001	3.15 0.009	3.18 0.009	3.15 0.01	2.48 0.007	3.91 0.01

## Discussion

Our study highlights the interaction between olive cultivar susceptibility with the infestation of *Bactrocera oleae*, with a specific focus on the variability in wing morphology. Conducted at 2 sites, Banjevci and Vodnjan, our research showed distinct levels of infestation, emergence, and morphological adaptation. The higher humidity levels observed in Vodnjan supported increased numbers of flies, suggesting that even slight microclimatic differences can significantly impact the development of *B. oleae* in Mediterranean olive-growing areas (Chang et al. 2008, Pappas et al. 2011, Kalamatianos et al. 2019).

A clear relationship emerged between specific cultivar traits and pest susceptibility, with larger-fruited cultivars such as Oblica, Istarska bjelica, and Ascolana tenera experiencing higher infestation rates. This aligns with findings from previous studies, indicating that larger fruit surface areas and increased volatile emissions attract *B. oleae* females to these cultivars for oviposition (Dminić et al. 2007, Daane and Johnson 2010, Karakoyun and Uçkun 2023).

In contrast, cultivars with smaller fruits, like Frantoio and Leccino, showed lower infestation levels, likely due to smaller fruit size, which makes them less attractive or suitable for oviposition (Škarica et al. 1996, Bulimbašić 2011). Similar studies in the Istria region (Dminić et al. 2007) showed analogous results, with the Istarska bjelica cultivar having a very high *B. oleae* infestation (97%), while the Leccino cultivar was significantly less affected (16%). This reinforces the idea that physical fruit traits, beyond purely weather conditions, influence the pest’s oviposition behavior. These findings suggest that the selection of certain olive cultivars could serve as a passive control strategy, reducing pest infestation pressure through cultivar-specific resistance traits.

A significant number of *B. oleae* specimens developed in visually uninfested fruit, suggesting that visual inspection alone is not sufficient to accurately assess the extent of infestation in the field. Research by Genc (2014) has shown that injuries to the olive fruit caused by female oviposition do not necessarily lead to successful oviposition.

Our geometric morphometric analysis, focusing on wing shape, provided further evidence of *B. oleae*’s adaptability, revealing pronounced sexual dimorphism in wing morphology. Female flies consistently displayed broader wings than males, a feature commonly associated with dispersal needs and oviposition behaviors in different insect orders such as Diptera, Lepidoptera, Hymenoptera, and Coleoptera (Bonduriansky 2006, Gidaszewski et al. 2009, Marsteller et al. 2009, Benítez et al. 2011, 2013, Lemic et al. 2014, 2020, 2021a, b, Le Roy et al. 2019). Wing shape variation was most prominent among females of cultivars like Frantoio and Oblica, suggesting adaptive responses that may optimize female mobility and efficiency in locating suitable oviposition sites on larger fruits. This sexual dimorphism likely reflects evolutionary pressures favoring enhanced dispersal and host selection capabilities in females, supporting previous findings that link morphological traits to behavioral and reproductive strategies in pest species (Hendrichs et al. 2002, Benelli 2014). In the case of *B. oleae*, this sexual dimorphism influenced the positioning of landmarks 12, 16, 17, and 18, which correspond to the radial, medial, and anal veins, crucial anatomical features for distinguishing wing morphotypes among tephritids (Van Cann et al. 2015). Female *B. oleae* in the populations of both regions showed broader wings compared to males. Elongated wings are typically associated with species or sexes involved in migratory movements. Accordingly, this study provides morphological evidence suggesting non-dispersive movements in adult *B. oleae*.

The observed overlap in wing shapes among certain cultivars across locations points to a high degree of phenotypic plasticity in *B. oleae*, enabling it to exploit different host sizes and fruit qualities under diverse cultivation conditions (for the medfly in Lemic et al. 2021b). This plasticity is particularly relevant in the context of different agricultural systems, as seen in the organic and integrated production at the Banjevci and Vodnjan sites. Such adaptability may have implications for pest resilience, as it could allow *B. oleae* to quickly adjust to changing cultivation pressures, underscoring the importance of cultivar selection as an adaptive pest management strategy (Letourneau and Bothwell 2008). Future studies could explore this plasticity further to determine the extent to which weather conditions or cultivar traits drive morphological changes.

Overall, this study demonstrates the utility of geometric morphometrics in capturing subtle, cultivar-specific morphological adaptations in *B. oleae*, enriching our understanding of pest-cultivar interactions.
